# Case of Enterotomy

**Published:** 1846-10

**Authors:** G. Conger


*Case of Enterotomy.* Reported by G. Conger, M. D.—Jeremiah Brown, aged 26, a strong muscular man, of good habits, on the 21st of May last received a stab in the abdomen with a large butcher's knife. The knife entered the abdomen one and a half inches from the ant. sup. spinous process of the ileum, on the left side, in a line from that process to the umbilicus: cutting upward, or nearly upward, to the extent of three and a fourth inches.

I saw him one hour and a half after the injury was inflicted, and found him faint from loss of blood; pulse 90, feeble; a large mass of intestines protruding from the wound covered with coagulated blood, and the bed saturated from profuse hemorrhage. After washing away the coagula, I found the omentum divided in such a manner as to leave a strip four or five inches long, and varying in width from one and a half inches at the lower end, to half an inch at the upper part, where it hung from the main body of omentum. This strip, or portion, I divided with the scissors at its narrow point, and removed. On farther examination, I found the arch of the *colon* nearly severed at a point, as near as I could judge, of some eight or ten inches from the cæcum. As the knife entered with the edge upward, the point penetrated the colon at its lower edge or surface, where the peritoneum doubles upon itself after surrounding the bowels to form the mesentery, so that the mesentery escaped, while the intestine was completely severed, save a narrow strip were the mesentery unites with the intestine, of three eighths of an inch in width. I also found a wound in the colon six inches higher up, which was a longitudinal slit, or puncture in the gut, of an inch in length. There were other slight scratches on the protruded intestines and omentum.

On determining the amount of injury, I was at a loss as to what I should do. My first impression was that he must certainly die, and that if any thing could be done towards a cure, it would be by bringing down the upper part of the intestine, return the mass, and try to form an artificial anus. But on consulting a few minutes with my friend Dr. Cook, and an intelligent gentleman from Black Rock, and laying before them the improbability of success in any operation we could perform, I finally determined to unite the intestine by suture and return it. After tying four small arteries that were bleeding from the wounded intestine, I brought the divided edges of the gut into as perfect apposition as possible, and fastened them by four interrupted sutures with common sewing silk, cutting off the ends of the ligatures close to the knots. Some feculent matter was discharged from the wound, but this, as well as all the hemorrhage, was external; in fact I do not believe a teaspoonful of blood was discharged into the cavity of the abdomen. Leaving the smaller wound of the colon, we returned the mass, and finished the operation by bringing the external wound together with three interrupted sutures, and adhesive strap, then applying a compress which we secured with a wide bandage. Gave him tr. opii. gt. 40, flexed the thighs upon the body, supported the legs with pillows under the knees, and left him for three or four hours.

I went to see him again at 5 o'clock, P. M., 8 hours after the injury; complained of severe pain in the region of the umbilicus; extremities rather cold; countenance anxious; pulse 92; soft and feeble. Has a desire to pass urine, but feels a total loss of power in the muscles of the abdomen, and can make no effort. Introduced the catheter and drew away twelve ounces. Gave, again, tr. opii. gt. xxv. Staid all night with him.

May 22d, 8 o'clock, A. M. Passed a restless night; pulse 98 and hard; abdomen tympanitic, and very tender to the touch; severe pain at the precordia; constant and distressing nausea. Drew off a pint of urine; took 16 oz. blood; pulse came down to 90, and became soft. Gave him tr. opii. gt. xx; directed him to take nothing but mucilaginous drinks. 7 o'clock, P.M. Tongue slightly furred; considerable thirst; had vomited a little, but nothing except his drink; tympanitis increasing; pulse hard; countenance flushed; skin hot and dry. Took 12 oz. blood, which produced fainting. Emptied the bladder, and gave, again, tr. opii. gt. xxv.

May 23d, 8 o'clock, A. M. Passed a more comfortable night; pain at intervals in the region of the wound; tongue dry and red; tympanitis, nausea, vomiting, and a tendency to hiccough; countenance anxious; pulse 98, small and wiry; a little flatus passed the bowels; external wound discharged a quantity of sanies. Continued tr. opii. gt. xx, with mucilages, and a little lemon juice, as a common drink. 6 o'clock, P.M. Had vomited several times; some hiccough; urine scanty. Treatment as before. Emptied the bladder three times a day.

May 24th, 8 o'clock, A. M. Countenance looks better; tongue red and a little shining; less pain and tympanitis; considerable flatus passed the bowels; pulse 90, full and hard. Took 10 oz. of blood; vomiting and hiccough ceased. Treatment as before. 7 o'clock, P. M. All the symptoms were favorable. Treatment the same.

May 25, 8 o'clock, A. M. Symptoms decidedly better; tongue moist; pulse 84. This being the fifth day since the acccident and there having been no movement of the bowels, I gave an enema, which, after being repeated, brought away hardened fæces, without pain. Ordered him the following ℞ Ol. Ricini, ℥ ii., Ol. Croton, gt. ii., mix and divide into three doses, one to be taken every four hours, unless the bowels should move. 7 o'clock, P. M. Bowels moved slightly about an hour before I arrived ; small quantity of grumous blood in the passage ; little pain attended the stool. Directed him to take sal. epsom, ℥ ss., at bedtime.

May 26th, 11 o'clock, A. M. Had two rather scanty dejections in the morning, some griping ; feels better ; pulse 75 ; tympanitis and tenderness fast subsiding. Gave him boiled rice and milk diet.

May 27th, 12 o'clock, M. Passed a good night, free from pain ; bowels moved without pain ; stool natural. Passed urine without the catheter. Pulse down to 65. Directed animal broths, toast, &c.

May 28th, 12 o'clock, M. Feels well ; some appetite ; pulse 62. Sediment in urine ; had a natural and free passage from the bowels. External wound looks pale and flabby ; removed the sutures ; and dressed it with spirits turpentine and adhesive strap.

May 29th, 12 o'clock, M. Same as yesterday ; pulse 58 ; appetite good. Gave him stimulating and nourishing diet.

May 30th, 12 o'clock, M. Pulse up to 65 and all other symptoms improving.

May 31st, 12 o'clock, M. Pulse 68 ; continued to improve rapidly until the 5th inst., when I ceased my visits. I have occasionally seen him since, and he has been down to see me (a distance of five miles.)

I have been perhaps tedious in detailing the above, but as it is a case of rare occurrence, and one of much importance in a surgical point of view, I felt that a statement of the case was demanded. The result has also been so favourable, that I deem the circulation of it among my medical brethren, will tend to advance our science, and correct some erroneous opinions in regard to the union of *divided intestines.*

In connection with the case, I would make a few remarks on *Enterotomy* or *Enteroraphia*, and its results, as it has been practised in this country and in Europe.

Few cases of this kind have occurred to give us much experimental knowledge on the subject of uniting divided intestines, either by suture or otherwise, in the human species. It is said of Ramdohr that he cut away a portion of mortified intestine, and joined the two ends by slipping the upper into the lower one, fixing them with a suture, and the patient did well. This is doubted by modern surgeons, from experiments made upon animals, for they find the mucous and serous surfaces do not unite, and the animals invariably die.

Dr. Smith, of Philadelphia, tried three experiments upon dogs by dividing an intestine, uniting it again with *one stitch*, and bringing the ends of the ligature out of the external opening. They all died from extravasation of fæces and blood in the abdomen.

Mr. Travers tried the same experiment, but failed. He tried another and used three stitches in the bowel, but the dog died.

From these experiments, therefore, this gentleman was led to believe and maintain that the *absolute contact* of the everted surfaces of a divided intestine in their *entire circumference,* is requisite to secure the animal from danger of abdominal effusion.

In opposition to this doctrine are the experiments of Sir Astley Cooper, Dr. Thompson, of Edinburgh, and others, where the intestine of a dog was divided, and united, some with three, others with four and five interrupted sutures, and were successful; but they cut the ends of the ligature close to the knot, and closed the external opening, as first recommended by Benjamin Bell.

In the case I have narrated, I adopted the plan recommended by Sir Astley, Dr. Thompson, Mr. Bell and others, in opposition to that of Mr. Travers, and some modern surgeons, in the belief that the *fewer the stitches taken,* and the less done to irritate so sensitive and delicate a structure as the peritoneum (after securing a proper coaptation of the parts), the greater would be the chances of recovery. You will also see from the detail of the case, that there was no extravasation of fæces, or effusion of blood into the cavity of the abdomen. In this case my opinion is, that the wounded intestine united by the *first intention,* as there was no discharge of purulent matter in the evacuations. I watched the stools carefully until the tenth day, expecting to see the sutures come away with the dejections, but was not fortunate enough to find them. There being eight in all (four sutures and four ligatures to the bleeding arteries), I supposed I should find the knots as they loosened, fell into the canal and were carried off, but I was disappointed.—*Buffalo Med. Jour.*

